# Production performance of four lines of Japanese quail reared under tropical climatic conditions of Tamil Nadu, India

**DOI:** 10.3389/fgene.2023.1128944

**Published:** 2023-04-05

**Authors:** Keche Vishal Arunrao, Duraisamy Kannan, Ramasamy Amutha, Aranganoor Kannan Thiruvenkadan, Abdulmojeed Yakubu

**Affiliations:** ^1^ Department of Poultry Science, Veterinary College and Research Institute, Namakkal, Tamil Nadu, India; ^2^ Department of Animal Genetics and Breeding, Veterinary College and Research Institute, Salem, Tamil Nadu, India; ^3^ Centre for Sustainable Agriculture and Rural Development, Department of Animal Science, Faculty of Agriculture, Nasarawa State University, Keffi, Nigeria

**Keywords:** Japanese quails, breeding lines, production traits, livability, tropics

## Abstract

This research investigated the growth and other production traits of four distinct lines (L1, L2, L3, and L4) of Japanese quail (Cortunix japanoica) kept in the tropical climate of Tamil Nadu, India. The traits related to body weight at different weeks and weight gain were measured in 180 birds (90 males and 90 females) per line up to the fifth week of age, and then 90 birds (females only) from the sixth to the 16th week of age, with egg production and feed efficiency parameters measured in 10 observations per line. The traits were analysed using the General Linear Model procedure, and Tukey’s HSD was used to test for statistical differences (*p* < 0.05) between the means for subclasses under a specific effect. The results revealed a highly significant difference (*p* < 0.01) in body weight from hatch to fifth week of age. At 5th week of age, the L3 and L2 did not differ (*p* > 0.05) based on Tukey test; therefore, both presented the highest values and were statistically significant (*p* < 0.01) with L4 with a lowest value of 203.62 g. The cumulative fifth week feed conversion ratio showed non-significant difference (*p* > 0.05) at first and second week, and highly significant difference (*p* < 0.01) from third to fifth week of age. The age at 50 per cent egg production was 60.2 (L4), 61.4 (L2), 65.1 (L3) and 66.0 (L1) days and the L1 egg production differed significantly (*p* < 0.01) from L4 line. The highest bodyweight (g) during the laying period (at 15 weeks of age) was observed in L2 (327.08) and L3 (326.54) and differed significantly (*p* < 0.01) from L1 (309.24) as well as L4 (288.69) lines. The mean egg weight (g) of different lines showed non-significant difference (*p* > 0.05) at all weeks, except at 11th week of age (*p* < 0.01). The mean feed consumption (g)/bird/day differed significantly (*p* < 0.01) from 6th to 16th week of age, except at sixth and eighth week of age, where it was non-significant (*p* > 0.05). The overall feed efficiency/dozen of eggs (from 6th to 16th weeks) ranged from 1.33 (L1) to 1.98 (L3). The livability from 6 to 16 weeks of age was 100 per cent in all the lines. In order to boost Japanese quail production in the tropics, L3 and L4 may be selected for body weight and egg production, respectively.

## 1 Introduction

Poultry production is one of agriculture’s fastest growing subsectors, producing a variety of commodities for the world’s population. Broiler chickens, commercial layers, turkeys, ducks, and quails are common meat and egg producers. These high-yielding birds have been genetically improved to have higher growth and egg-laying rates. The goal of developing such strains was to meet the global population’s dietary needs, particularly for proteins (Hussain et al., 2018; Ghayas et al., 2020). The Japanese quails (*Coturnix japonica*) are the smallest possible avian species raised for meat and egg production. Several factors contribute to their utility and cost-effective alternative to chicken’s production (Kayang et al., 2004; Sezai et al., 2010), such as: shorter production cycle, early maturity and lower initial investment required for the commercially rearing of quails. In addition, they are associated with healthier meat and eggs (Ghayas et al., 2017; Ahmad et al., 2018), present a meat with a distinct flavor and high nutritional value (Kayang et al., 2004), and the resource poor people all over the world are interested in commercially rearing quails due to the lower initial investment. Many meat consumers prefer quail meat because of its low fat content, primarily saturated fatty acid and cholesterol level, when compared to similar cuts of red meat such as beef and mutton (Boni et al., 2010) and white meat such as broiler chicken and duck (Ionita et al., 2008). According to Genchev et al. (2008), a daily intake of two quails provides the human body with 27–28 g protein, 11 g of essential amino acids, and covers 40 per cent of the human protein requirement.

In general, the production performance of the Japanese quail can be improved by increasing their genetic potential and providing favourable management conditions. One of the most crucial economic variables in any genetic improvement programme is body weight, along with egg production, for a number of reasons, including its relationship to other meat production traits and their relative simplicity of measuring. The true economic and commercial value of this amazing bird is found in its egg production, as domesticated lines of Japanese quail can lay up to 290 to 300 eggs in their first year of lay (Jatoi et al., 2015). At the age of 4 weeks, it has the potential to gain 160–170 g of body weight (Akram et al., 2014). The fundamental tool for maximising the productive potential of birds is by selection. Around the world, a variety of selection techniques have been in use, ranging from mass selection to complete pedigree selection (Krishna et al., 2016; Narinç et al., 2016; Durmuş et al., 2017; Pandian et al., 2017) to improve the Japanese quails’ performance.

Quail farming is a growing industry in India, because of the relatively higher profit margins (Pandian et al., 2017; Prabakaran and Ezhil Valavan, 2020). As there is a need to promote the growth of these birds through selection programmes, different production performances were utilized for the genetic selection in the four different lines (L1, L2, L3, and L4) of Japanese quail maintained at the Poultry Farm Complex of Department of Poultry Science, Veterinary College and Research Institute, Namakkal, Tamil Nadu, India for the production of parent breeders and the characteristics that are taken into account are body weight, growth rate and egg production. Selection experiments provide background knowledge of complex trait inheritance and allow for the evaluation of hypothetical predictions by comparing observations to expectations. Long-term response to selection is more focused on fixing the probabilities of alleles responsible for the trait(s) under consideration and short-term selection response, on the other hand, could be attributed to segregating alleles in the population (Fuller et al., 2005). Therefore, the present study was conducted to evaluate the production performance of four different lines of Japanese quail maintained under the tropical climatic conditions of India, and to select the best lines for different purposes including commercial production for economic rearing for the betterment of smallholder farming communities.

## 2 Materials and methods

### 2.1 Study area and genetic information of the lines

The experiment was conducted at the Poultry Farm Complex, Department of Poultry Science, Veterinary College and Research Institute, Namakkal, This complex is located at an elevation of 192 m above mean sea level and at 11°2′N latitude and 78°2′ E longitudes. The average minimum and maximum temperatures as well as the relative humidity were 20.39°C and 33.64°C and 46.94 and 87.27 per cent, respectively. For this study, four Japanese quail lines, L1, L2, L3, and L4 were used and lines were genetically unrelated and purchased from different sources to maximise heterosis in the year 2005. The lines L1, L2, and L4 were obtained from three different quail breeding companies located in different parts of Southern India, while the L3 line was obtained from a quail breeder cum farmer in Tamil Nadu. The lines were maintained for several generations and subjected to genetic selection for improving the meat and egg production with different traits, which would include L1 for egg weight, L2 for age at 50% egg production and medium body weight, L3 for higher body weight at 6 weeks of age, and L4 for egg number.

### 2.2 Experimental design and birds’ management

Hatching eggs from four different lines of Japanese quail, namely, L1, L2, L3, and L4, were incubated separately, and the chicks that hatched were used in the study. The experiment was divided into two parts: growth performance of four lines of Japanese quail from 0 to 5 weeks of age, and growth and egg production performance of four lines of Japanese quail from 6 to 16 weeks of age. The birds were used as parent stock for the production of day-old chicks for the experiment when they were 16 weeks old. A total of 20 males and 80 females were randomly selected from each line (L1, L2, L3, and L4), and half-sib mating was used within each line, with one male and four females of selected birds housed together in each breeding cage. Eggs were collected separately and stored in a cold room before being placed in an incubator to hatch. After hatching, the 180 day-old straight-run Japanese quail chicks from each line were weighed individually and immediately transferred to the brooding cages. A total of 18 chicks per cage were reared up to 2 weeks of age in the brooding cages. From third week of age, birds were transferred to grower cages and kept up to 5 weeks of age. The birds were wing banded at third week for identification and proper recording of production data. After the completion of the growth study, ninety females from each line were taken and reared in layer cages by keeping nine females per cage up to 16 weeks of age to study the growth and egg production performances. The traits such as body weight, body weight gain, cumulative feed conversion ratio were recorded up to fifth week of age in both males and females and then to study the laying performances the traits *viz.*, age (days) at first egg, age at 50 per cent egg production, age at 90 per cent egg production, hen-housed egg production, hen-day egg production, egg weight, feed consumption (g)/bird/week and weekly feed efficiency/dozen eggs were recorded. At each experimental stage, the quails were given weighed amounts of feed to eat *ad libitum*, along with unrestricted access to clean water. The overall amount of light exposure per day was 16 hours. Other routine management practices were carried out.

### 2.3 Data collection

During the growth period, body weight at weekly interval, feed consumption and egg production on daily basis and mortality, if any, were recorded during the laying period. The weight of the eggs was measured once a week; in addition, age at sexual maturity, hen-day egg production, hen-housed egg production, livability, and feed efficiency in terms of feed per dozen eggs were calculated. To assess production performance, the individual weights of all chicks from four lines (L1, L2, L3, and L4) were recorded at the 0^th^ day (hatch weight), first, second, third, fourth and fifth week of age, and subsequently measured at fortnightly intervals from the 7th to 15th week of age. Throughout the study, the daily feed consumption (g) per bird was recorded and the average feed consumption per bird was calculated at the end of each week by subtracting the leftover feed from the total amount of feed given. The feed conversion ratio was calculated by dividing the amount of feed consumed by the bird by the amount of weight gain. The individual egg weight (g) was recorded with an electronic balance with an accuracy of 0.001 g. The Hen-day egg production was calculated by using the following formula.

$$Hen-day\;egg\;production\;for\;the\;period=\frac{Number\;of\;eggs\;laid\;during\;the\;period}{Sum\;of\;number\;of\;hens\;survived\;per\;day\;for\;the\;period}\times 100$$

The Hen-housed egg production (eggs per bird) from 6th to 16th weeks of age was calculated by

$$Hen\;housed\;egg\;production\;for\;the\;period=\frac{Average\;number\;of\;eggs\;produced\;during\;the\;period}{Total\;number\;of\;hens\;housed\;in\;the\;beginning}\times 100$$

The feed efficiency was expressed in kilograms of feed consumed to produce one dozen eggs. The total feed consumed (kg) during 6–16 weeks of age was calculated in all the four lines. The total number of eggs produced during the respective periods was arrived by adding the number of eggs produced during the period for each experimental line. The feed efficiency was calculated as follows:

$$Feed\;efficiency\;\left(per\;dozen\;of\;eggs\right)=\frac{Total\;kilograms\;of\;feed\;consumed\;during\;the\;period}{Total\;number\;of\;eggs\;produced\;during\;the\;period}\times 12$$

Mortality was recorded as at occurrence during the study period and breeder house livability (%) was calculated by using the following formula.

$$Livability=\frac{Total\;number\;of\;live\;birds\;during\;the\;period}{Total\;number\;of\;birds\;present\;in\;the\;breeder\;house\;at\;the\;beginning\;of\;study}\times 100$$

### 2.4 Statistical analysis

In order to ascertain the normality of the data and the residuals, they were subjected to Shapiro-Wilk’s test (*p* > 0.05), while homogeneity of variances (*p* > 0.05) was confirmed using Levene’s test as described by Yakubu et al. (2022). The traits such as body weight, body weight gain, cumulative feed conversion ratio, age (days) at first egg, age at 50 per cent egg production, age at 90 per cent egg production, hen-housed egg production, hen-day egg production, egg weight, feed consumption (g)/bird/week and weekly feed efficiency/dozen eggs were analyzed using General Linear Model procedure of Statistical Package for Social Sciences (SPSS) 25th version (IBM Corp, 2019) and the statistical model used for the analysis was Y_ij_ = µ + L_i_ + e_ij_ (where Y_ij_ is the observed production and reproduction parameters analysed individually; µ = Overall mean; L_i_ = effect of *i*th line (i = 1–4) and e_ij=_random errors). Statistical differences (*p* < 0.05) between the means for subclasses under a particular effect were tested by Tukey’s HSD test.

## 3 Results

### 3.1 Body weight and body weight gain from hatch to fifth week

Table 1 shows the means (±S.E.) of body weight (g) and body weight gain (g/week) of various lines of Japanese quail from hatch to fifth week of age. The body weight of the four lines of Japanese quail differed significantly (*p* < 0.01) from hatch to the fifth week of age. However, the difference between lines was not uniform and varied with different ages. Highest fifth week body weight was recorded in L3 (235.31 g) and L2 (232.99 g) and did not differ significantly (*p* < 0.01) with them but both differed significantly (*p* < 0.01) from L1 (217.05 g) and L4 (203.62 g). From hatch to the fifth week of age, the average body weight gain of L2 (223.97 g) and L3 (226.07 g) was substantially identical and non-significant (*p* > 0.05), but they differed significantly (*p* < 0.01) from L1 (208.06 g) and L4 (194.29 g).

**TABLE 1:** Means (±S.E.) of body weight (g) of different lines of Japanese quail from hatch to fifth week of age.

Effect	Number of observations	Body weight (g)						Body weight gain (g)/week					
		Hatch weight	First week	Second week	Third week	Fourth week	Fifth week	First week	Second week	Third week	Fourth week	Fifth week	Overall (hatch to fifth week)
**Overall**	**720**	9.15 ± 0.02	27.35 ± 0.07	67.15 ± 0.14	114.32 ± 0.52	177.27 ± 0.66	222.25 ± 0.73	18.20 ± 0.06	39.80 ± 0.20	47.17 ± 0.52	62.95 ± 0.36	44.98 ± 0.44	213.10 ± 0.72
**LINE**		∗∗	∗∗	∗∗	∗∗	∗∗	∗∗	∗∗	∗∗	∗∗	∗∗	∗∗	∗∗
Line 1	**180**	8.99^a^ ± 0.05	28.32^c^ ± 0.14	67.52^b^ ± 0.28	113.85^b^ ± 1.04	172.36^a^ ± 1.33	217.05^b^ ± 1.45	19.33^c^ ± 0.11	39.80^ab^ ± 0.41	46.33^b^ ± 1.04	58.51^a^ ± 0.72	44.69^b^ ± 0.88	208.06^b^ ± 1.44
Line 2	**180**	9.02^a^ ± 0.05	27.34^b^ ± 0.14	67.42^b^ ± 0.28	119.16^c^ ± 1.04	182.63^b^ ± 1.33	232.99^c^ ± 1.46	18.32^a^ ± 0.11	40.08^b^ ± 0.41	51.74^c^ ± 1.04	63.47^b^ ± 0.72	50.36^c^ ± 0.88	223.97^c^ ± 1.44
Line 3	**180**	9.24^b^ ± 0.05	27.34^b^ ± 0.14	67.60^b^ ± 0.28	117.17^bc^ ± 1.05	186.48^b^ ± 1.33	235.31^c^ ± 1.46	18.10^a^ ± 0.11	40.26^b^ ± 0.41	49.57^bc^± 1.04	69.31^c^ ± 0.72	48.83^c^ ± 0.88	226.07^c^ ± 1.44
Line 4	**180**	9.33^b^ ± 0.05	26.41^a^ ± 0.14	66.05^a^ ± 0.28	107.10^a^ ± 1.04	167.60^a^ ± 1.33	203.62^a^ ± 1.46	17.08^a^ ± 0.11	39.64^a^ ± 0.41	41.05^a^ ± 1.04	60.50^a^ ± 0.72	36.02^a^ ± 0.88	194.29^a^ ± 1.44
**F value**		11.87	33.19	6.96	25.95	43.66	103.29	70.66	9.02	17.68	44.41	40.99	96.86
***p*-value**		0.00	0.00	0.00	0.00	0.00	0.00	0.00	0.00	0.00	0.00	0.00	0.00

Means with at least one common superscript within age group do not differ significantly (*p* ≥ 0.05).

^a^ Highly Significant (*p* < 0.01).

### 3.2 Feed consumption and feed conversion ratio from 0 to fifth week of age

Table 2 depicts the mean (±S.E.) of the cumulative feed conversion ratio for different lines of Japanese quail from the first to fifth week of age. The feed conversion ratio observed between the lines were non-significant (*p* > 0.05) in the first and second week, but differed significantly (*p* < 0.01) in subsequent weeks with varied response. At three and 5 weeks of age, the L1, L2, and L3 generally had feed conversion efficiencies that were nearly similar and did not differ significantly (*p* > 0.05) from one another. These three lines also had higher feed conversion efficiencies than L4 and differed significantly (*p* < 0.01). Among the four lines evaluated, the L4 feed conversion efficiency was low.

**TABLE 2:** Means (±S.E.) of cumulative feed conversion ratio of different lines of Japanese quail from first to fifth week of age.

Effect	Number of observations	Age in weeks				
		1	2	3	4	5
**Overall**	**40**	2.04 ± 0.03	2.37 ± 0.01	2.78 ± 0.02	2.83 ± 0.01	3.24 ± 0.02
**LINE**		**NS**	**NS**	**	**	**
Line 1	10	1.93 ± 0.07	2.36 ± 0.03	2.73^a^ ± 0.04	2.87^bc^ ± 0.02	3.22^a^ ± 0.03
Line 2	10	2.02 ± 0.07	2.36 ± 0.03	2.69^a^ ± 0.04	2.79^ab^ ± 0.02	3.14^a^ ± 0.03
Line 3	10	2.12 ± 0.07	2.37 ± 0.03	2.76^a^ ± 0.04	2.74^a^ ± 0.02	3.16^a^ ± 0.03
Line 4	10	2.11 ± 0.07	2.40 ± 0.03	2.94^b^ ± 0.04	2.92^c^ ± 0.02	3.43^b^ ± 0.03
**F value**		1.72	0.56	8.41	11.44	16.88
***p*-value**		0.18	0.64	0.00	0.00	0.00

Means with at least one common superscript within age group do not differ significantly (*p* ≥ 0.05).

NS Not, significant **Highly Significant (*p* < 0.01).

### 3.3 Body weight of females of different lines at the laying period

The mean body weight of breeder female quail lines increased progressively from the 7th to 11th week of age (Table 3). Later, the increase was meagre up to 13th week as well as reduction of body weight was noticed at 15th week of age. At the 13th week of age, body weight obtained in L2 and L3 were not different (*p* > 0.05) from each other, but they were higher than that observed for L1 and L4 lines. A similar pattern was observed at the 15th week of age. In all the lines, the body weight at the 15th week was slightly lower than the values observed at the 13th week of age.

**TABLE 3:** Means (±S.E.) of body weight (g) of different lines of Japanese quail layers from 7th to 15th week of age.

Effect	Number of observations	Age in weeks				
		7	9	11	13	15
**Overall**	**360**	266.87 ± 1.51	308.90 ± 1.59	319.11 ± 1.72	319.23 ± 1.65	312.89 ± 1.67
**LINE**		^**∗∗**^	^**∗∗**^	^**∗∗**^	^**∗∗**^	^**∗∗**^
Line 1	90	252.99^a^ ± 3.01	297.04^a^ ± 3.19	310.89^b^ ± 3.43	316.85^b^ ± 3.30	309.24^b^ ± 3.33
Line 2	90	270.01^b^ ± 3.01	319.13^b^ ± 3.19	326.82^c^ ± 3.43	333.52^c^ ± 3.30	327.08^c^ ± 3.33
Line 3	90	286.25^c^ ± 3.01	329.36^b^ ± 3.19	341.44^d^ ± 3.43	333.60^c^ ± 3.30	326.54^c^ ± 3.33
Line 4	90	258.23^a^ ± 3.01	290.06^a^ ± 3.19	297.27^a^ ± 3.43	292.94^a^ ± 3.30	288.69^a^ ± 3.33
**F value**		24.01	33.44	31.23	33.94	29.66
***p*-value**		0.00	0.00	0.00	0.00	0.00

Means with at least one common superscript within age group do not differ significantly (*p* ≥ 0.05).

^a^ Highly Significant (*p* < 0.01).

### 3.4 Age at sexual maturity and egg production parameters

For the four lines of Japanese quail, the mean (±S.E.) of age (days) at first egg, age at 50 and 90 per cent of egg production are shown in Table 4. For the age of sexual maturity as determined by the first egg deposited, there was no statistically significant (*p* > 0.05) difference between the four lines. However, the age at which 50 per cent of eggs produced, revealed a significant variability (*p* < 0.01) with the earliest production occurring in L4 (60.2 days) and differed significantly only with L1 (66.0 days) line. There was no statistically significant (*p* > 0.05) difference in the age at which 90 per cent of egg production between the lines.

**TABLE 4:** Means (±S.E.) of age (days) at first egg, age at 50 per cent and 90 per cent egg production of different lines of Japanese quail.

Effect	Number of observations	Age at first egg	Age at 50% egg production	Age at 90% egg production
**Overall**	**40**	50.78 ± 0.88	63.18 ± 0.74	75.88 ± 1.29
**LINE**		**NS**	*	**NS**
Line 1	**10**	51.70 ± 1.76	66.00^b^ ± 1.48	78.90 ± 2.58
Line 2	**10**	52.50 ± 1.76	61.40^ab^ ± 1.48	78.40 ± 2.58
Line 3	**10**	51.00 ± 1.76	65.10^ab^ ± 1.48	74.20 ± 2.58
Line 4	**10**	47.90 ± 1.76	60.20^a^ ± 1.48	72.00 ± 2.58
**F value**		1.30	3.63	1.68
***p*-value**		0.29	0.02	0.19

Means with at least one common superscript within the economic traits do not differ significantly (*p* ≥ 0.05).

NS Not, significant *Significant (*p* < 0.05).

The overall hen-housed egg production pooled over 6th to 16th week of age (Table 5) was much higher in L4 (46.30) and L3 (44.24) and differed significantly (*p* < 0.01) from L1 (38.27), but there were no significant difference (*p* > 0.05) between L2, L3, and L4 as well as L1 and L2. The overall hen-day egg production (Table 6) observed for the L3 and L4 lines differed significantly (*p* < 0.01) from L1 line.

**TABLE 5:** Means (±S.E.) of hen-housed egg production (eggs per bird) of different lines of Japanese quail from 6th to 16th week of age.

Effect	N	Age in weeks											
		6	7	8	9	10	11	12	13	14	15	16	Overall (6–16)
**Overall**	**40**	0.01 ± 0.01	0.33 ± 0.07	0.92 ± 0.13	2.36 ± 0.16	4.07 ± 0.16	5.16 ± 0.13	5.43 ± 0.13	5.70 ± 0.14	6.04 ± 0.10	6.26 ± 0.09	6.42 ± 0.08	42.70 ± 0.69
**LINE**		**NS**	**NS**	*	**	**NS**	*	**	**	**	**NS**	**NS**	**
Line 1	10	0.00 ± 0.01	0.17 ± 0.14	0.67^ab^ ± 0.26	1.74^a^ ± 0.31	3.87 ± 0.32	4.57^a^ ± 0.25	4.82^a^ ± 0.25	4.90^a^ ± 0.28	5.22^a^ ± 0.20	5.97 ± 0.17	6.34 ± 0.15	38.27^a^ ± 1.38
Line 2	10	0.00 ± 0.01	0.30 ± 0.14	0.89^ab^ ± 0.26	2.33^ab^ ± 0.31	4.18 ± 0.32	4.95^ab^ ± 0.25	4.98^a^ ± 0.25	5.40^ab^ ± 0.28	6.24^b^ ± 0.20	6.33 ± 0.17	6.40 ± 0.15	42.00^ab^ ± 1.38
Line 3	10	0.02 ± 0.01	0.24 ± 0.14	0.56^a^ ± 0.26	1.91^a^ ± 0.31	3.66 ± 0.32	5.57^b^ ± 0.25	6.17^b^ ± 0.25	6.52^c^ ± 0.28	6.54^b^ ± 0.20	6.45 ± 0.17	6.60 ± 0.15	44.24^b^ ± 1.38
Line 4	10	0.01 ± 0.01	0.61 ± 0.14	1.58^b^ ± 0.26	3.44^b^ ± 0.31	4.59 ± 0.32	5.55^b^ ± 0.25	5.76^ab^ ± 0.25	5.76^bc^ ± 0.28	6.29^b^ ± 0.20	6.29 ± 0.17	6.32 ± 0.15	46.30^b^ ± 1.38
**F value**		0.73	1.85	3.19	6.08	1.63	3.68	6.54	6.30	8.09	1.51	0.69	6.18
***p*-value**		0.54	0.16	0.04	0.00	0.20	0.02	0.00	0.00	0.00	0.23	0.56	0.00

Means with at least one common superscript within age group do not differ significantly (*p* ≥ 0.05).

NS Not, significant *Significant (*p* < 0.05) **-Highly Significant (*p* < 0.01).

**TABLE 6:** Means (±S.E.) of hen-day egg production (%) of different lines of Japanese quail from 6th to 16th week of age.

Effect	N	Age in weeks											Overall (6–16)
		6	7	8	9	10	11	12	13	14	15	16	
**Overall**	**40**	0.12 ± 0.09	4.72 ± 1.03	13.17 ± 1.83	33.69 ± 2.22	58.17 ± 2.28	73.69 ± 1.81	77.58 ± 1.78	81.47 ± 2.00	86.31 ± 1.43	89.45 ± 1.21	91.67 ± 1.08	61.39 ± 1.88
**LINE**		**NS**	**NS**	*	**	**NS**	*	**	**	**	**NS**	**NS**	**
Line 1	10	0.00	2.38 ± 2.05	9.52^ab^ ± 3.67	24.92^a^ ± 4.43	55.24 ± 4.55	65.24^a^ ± 3.62	68.89^a^ ± 3.56	70.00^a^ ± 4.01	74.60^a^ ± 2.86	85.24 ± 2.42	90.64 ± 2.17	56.27^a^ ± 3.81
Line 2	10	0.00	4.29 ± 2.05	12.70^ab^ ± 3.67	33.33^ab^ ± 4.43	59.68 ± 4.55	70.79^ab^ ± 3.62	71.11^a^ ± 3.56	77.14^ab^ ± 4.01	89.05^b^ ± 2.86	90.48 ± 2.42	91.43 ± 2.17	61.76^ab^ ± 3.81
Line 3	10	0.32 ± 0.18	3.49 ± 2.05	7.94^a^ ± 3.67	27.30^a^ ± 4.43	52.22 ± 4.55	79.52^b^ ± 3.62	88.10^b^ ± 3.56	93.18^c^ ± 4.01	93.49^b^ ± 2.86	92.22 ± 2.42	94.29 ± 2.17	62.32^b^ ± 3.73
Line 4	10	0.16 ± 0.18	8.73 ± 2.05	22.54^b^ ± 3.67	49.21^b^ ± 4.43	65.56 ± 4.55	79.21^b^ ± 3.62	82.22^ab^ ± 3.56	85.56^bc^ ± 4.01	88.10^b^ ± 2.86	89.84 ± 2.42	90.32 ± 2.17	65.21^b^ ± 3.73
**F value**		0.73	1.84	3.19	6.08	1.62	3.67	6.55	6.32	8.11	1.51	0.70	0.98
***p*-value**		0.54	0.16	0.04	0.00	0.20	0.02	0.00	0.00	0.00	0.23	0.56	0.41

Means with at least one common superscript within age group do not differ significantly (*p* ≥ 0.05).

NS Not, significant *Significant (*p* < 0.05) **-Highly Significant (*p* < 0.01).

### 3.4 Egg weight, feed efficiency/dozen eggs and livability

The means (±S.E.) of egg weight (g) for various lines of Japanese quail from the 6th to 16th week of age are displayed in Table 7. The overall egg weight (g) from 6th to 16th week revealed that the L2 (12.77 g) differed significantly (*p* < 0.01) from L4 (12.36 g) only. The mean feed consumption/bird/week is presented in Table 8. The mean feed consumption (g)/bird/day differed significantly (*p* < 0.01) from 6th to 16th week of age, except at sixth and eighth week of age, where it was non-significant (*p* > 0.05). The means of weekly feed efficiency/dozen eggs (Table 9) indicated non-significant (*p* > 0.05) differences at weeks 7, 8, 10, and 15; significant differences (*p* < 0.05) at weeks 9, 13, and 16, and highly significant differences (*p* < 0.01) at weeks 11, 12, and 14. In general, after reaching 50 percent egg production, L4 (0.45) had the best feed efficiency and differed significantly (*p* < 0.01) from L3 (0.50), L2 (0.54), and L1 (0.57) lines. The livability observed from day-old to 16th week of age was 100 per cent.

**TABLE 7:** Means (±S.E.) of egg weight (g) of different lines of Japanese quail from 6th to 16th week of age.

Effect	N	Age in weeks											
		6	7	8	9	10	11	12	13	14	15	16	Overall (6–16)
**Overall**	**80**	9.62 ± 0.07	11.05 ± 0.11	11.84 ± 0.11	12.86 ± 0.25	12.36 ± 0.08	12.76 ± 0.05	12.82 ± 0.10	12.76 ± 0.73	12.73 ± 0.09	12.71 ± 0.08	12.94 ± 0.09	12.56 ± 0.04
**LINE**			**NS**	**NS**	**NS**	**NS**	**	**NS**	**NS**	**NS**	**NS**	**NS**	**
Line 1	20	-	10.83 ± 0.22	11.82 ± 0.22	12.35 ± 0.51	12.37 ± 0.17	12.83^b^ ± 0.10	12.88 ± 0.20	12.79 ± 0.15	12.42 ± 0.19	12.64 ± 0.16	12.80 ± 0.19	12.51^ab^ ± 0.07
Line 2	20	-	11.27 ± 0.22	12.02 ± 0.22	13.47 ± 0.51	12.47 ± 0.17	12.96^b^ ± 0.10	12.95 ± 0.20	12.85 ± 0.15	12.93 ± 0.19	12.84 ± 0.16	13.16 ± 0.19	12.77^b^ ± 0.07
Line 3	20	9.84 ± 0.48	11.06 ± 0.22	11.88 ± 0.22	12.98 ± 0.51	12.37 ± 0.17	12.82^b^ ± 0.10	12.84 ± 0.20	12.77 ± 0.15	12.88 ± 0.19	12.78 ± 0.16	12.94 ± 0.19	12.59^ab^ ± 0.07
Line 4	20	9.18 ± 0.00	11.05 ± 0.22	11.63 ± 0.22	12.63 ± 0.51	12.24 ± 0.17	12.43^a^ ± 0.10	12.62 ± 0.20	12.60 ± 0.15	12.66 ± 0.19	12.59 ± 0.16	12.84 ± 0.19	12.36^a^ ± 0.07
**F value**		-	0.68	0.55	0.91	0.34	4.95	0.53	0.56	1.60	0.58	0.76	5.36
***p*-value**		-	0.57	0.65	0.44	0.79	0.00	0.67	0.64	0.20	0.63	0.52	0.00

Means with at least one common superscript within age group do not differ significantly (*p* ≥ 0.05).

NS Not, significant **Highly Significant (*p* < 0.01).

**TABLE 8:** Means (±S.E.) of feed consumption (g)/bird/week of different lines of Japanese quail from 6th to 16th week of age.

Effect	N	Age in weeks											
		6	7	8	9	10	11	12	13	14	15	16	Overall (6–16)
**Overall**	**40**	205.65 ± 5.02	203.93 ± 3.09	251.92 ± 2.30	238.62 ± 2.03	223.53 ± 1.65	231.83 ± 1.62	224.53 ± 1.81	245.27 ± 3.31	222.62 ± 1.88	221.88 ± 1.85	203.25 ± 1.34	224.82 ± 1.09
**LINE**		**NS**	**	**NS**	**	**	**	**	*	**	**	**	**
Line 1	10	195.92 ± 10.03	179.35^a^ ± 6.17	244.93 ± 4.61	220.83^a^ ± 4.06	221.91^ab^ ± 3.29	233.82^b^ ± 3.24	227.92^b^ ± 3.63	249.73^ab^ ± 6.63	218.06^ab^ ± 3.76	213.18^a^ ± 3.70	198.08^a^ ± 2.67	218.52^a^ ± 2.19
Line 2	10	212.86 ± 10.03	216.54^b^ ± 6.17	257.67 ± 4.61	247.32^b^ ± 4.06	228.66^b^ ± 3.29	239.23^b^ ± 3.24	232.67^b^ ± 3.63	251.73^b^ ± 6.63	226.90^bc^ ± 3.76	223.92^ab^ ± 3.70	210.76^b^ ± 2.67	231.66^b^ ± 2.19
Line 3	10	221.94 ± 10.03	231.11^b^ ± 6.17	252.68 ± 4.61	245.95^b^ ± 4.06	231.59^b^ ± 3.29	235.15^b^ ± 3.24	227.47^b^ ± 3.63	253.79^b^ ± 6.63	234.23^c^ ± 3.76	237.16^b^ ± 3.70	214.63^b^ ± 2.67	235.06^b^ ± 2.19
Line 4	10	191.90 ± 10.03	188.73^a^ ± 6.17	252.38 ± 4.61	240.37^b^ ± 4.06	211.94^a^ ± 3.29	219.11^a^ ± 3.24	210.05^a^ ± 3.63	225.84^a^ ± 6.63	211.28^a^ ± 3.76	213.28^a^ ± 3.70	189.53^a^ ± 2.67	214.04^a^ ± 2.19
**F value**		1.99	15.17	1.30	9.10	7.03	7.34	7.5	3.88	7.15	9.42	18.71	21.41
***p*-value**		0.13	0.00	0.29	0.00	0.00	0.00	0.00	0.02	0.00	0.00	0.00	0.00

Means with at least one common superscript within age group do not differ significantly (*p* ≥ 0.05).

NS Not, significant **Highly Significant (*p* < 0.01).

**TABLE 9:** Means (±S.E.) of weekly feed efficiency/dozen eggs of different lines of Japanese quails from 6th to 16th week of age.

Effect	N	Age in weeks												
		6	7	8	9	10	11	12	13	14	15	16	After 50% egg production (10–16)	Overall (6–16)
**Overall**	**40**	21.27	9.17 ± 2.20	4.86 ± 1.26	1.53 ± 0.15	0.71 ± 0.04	0.56 ± 0.02	0.52 ± 0.02	0.55 ± 0.03	1.57 ± 0.20	0.43 ± 0.01	0.38 ± 0.01	0.51 ± 0.10	1.57 ± 0.20
**LINE**			**NS**	**NS**	*	**NS**	**	**	*	**	**NS**	*	**	**NS**
Line 1	10		9.02 ± 5.37	4.25 ± 2.43	2.07^b^ ± 0.30	0.75 ± 0.07	0.63^b^ ± 0.03	0.59^b^ ± 0.03	0.67^b^ ± 0.06	1.33 ± 0.41	0.43 ± 0.01	0.38^a^ ± 0.01	0.57^c^ ± 0.20	1.33 ± 0.41
Line 2	10		7.03 ± 4.65	6.30 ± 2.60	1.26^ab^ ± 0.31	0.71 ± 0.07	0.60^ab^ ± 0.03	0.59^b^ ± 0.03	0.59^ab^ ± 0.06	1.35 ± 0.42	0.44 ± 0.01	0.40^a^ ± 0.01	0.54^c^ ± 0.20	1.35 ± 0.42
Line 3	10	17.58	15.32 ± 3.80	3.43 ± 2.81	1.88^ab^ ± 0.30	0.83 ± 0.07	0.51^ab^ ± 0.03	0.44^a^ ± 0.03	0.47^ab^ ± 0.06	1.98 ± 0.41	0.44 ± 0.01	0.39^a^ ± 0.01	0.50^b^ ± 0.20	1.98 ± 0.41
Line 4	10	24.95	5.32 ± 3.52	5.46 ± 2.17	0.91^a^ ± 0.30	0.57 ± 0.07	0.49^a^ ± 0.03	0.44^a^ ± 0.03	0.46^a^ ± 0.06	1.60 ± 0.40	0.41 ± 0.01	0.36^a^ ± 0.01	0.45^a^ ± 0.20	1.60 ± 0.40
**F value**			1.35	0.23	3.36	2.32	4.54	6.02	3.51	4.51	0.54	3.11	6.72	0.54
***p*-value**			0.29	0.87	0.03	0.09	0.01	0.002	0.025	0.009	0.65	0.04	0.00	0.65

Means with at least one common superscript within age group do not differ significantly (*p* ≥ 0.05).

NS Not, significant *Significant (*p* < 0.05) **Highly Significant (*p* < 0.01).

In comparison between lines, the study found that the growth performance of L2 and L3 was higher in terms of maximum fifth week body weight and better feed conversion ratio, and hence both may be used to improve growth features (Table 10). The line L4 had a younger age of sexual maturity with superior egg production performance, including hen-day and hen-housed egg production, feed consumption, and feed efficiency per dozens of eggs. As a result, L4 line is better for reproductive performance, but a detailed repeated measures analysis with more samples is needed to make a meaningful judgment for utilizing the L4 lines for the improvement of reproduction parameters. The outcomes of the present study is in consonance with the selection criteria of the different lines, which included L2 for age at 50% egg production and medium body weight, L3 for greater body weight at 6 weeks of age, and L4 for egg number.

**TABLE 10:** Order of preference of the different lines for the different production and reproduction characters.

Parameters	Order of performance of lines			
**Body weight (5**th **week) (g)**	L3 (235.31^c^ ± 1.46)	L2 (232.99^c^ ± 1.46)	L1 (217.05^b^ ± 1.45)	L4 (203.62^a^ ± 1.46)
**Body weight of layers (15**th **week) (g)**	L2 (327.08^c^ ± 3.33)	L3 (326.54^c^ ± 3.33)	L1 (309.24^b^ ± 3.33)	L4 (288.69^a^ ± 3.33)
**Cumulative feed conversion ratio (5**th **week)**	L2 (3.14^a^ ± 0.03)	L3 (3.16^a^ ± 0.03)	L1 (3.22^a^ ± 0.03)	L4 (3.43^b^ ± 0.03)
**Feed efficiency after 50% egg production**	L4 (0.45^a^ ± 0.20)	L3 (0.50^b^ ± 0.20)	L2 (0.54^c^ ± 0.20)	L1 (0.57^c^ ± 0.20)
**Age at 50% egg production (days)**	L4 (60.20^a^ ± 1.48)	L2 (61.40^ab^ ± 1.48)	L3 (65.10^ab^ ± 1.48)	L1 (66.00^b^ ± 1.48)
**Egg weight (g)**	L2 (12.77^b^ ± 0.07 g)	L3 (12.59^ab^ ± 0.07 g)	L1 (12.51^ab^ ± 0.07 g)	L4 (12.36^a^ ± 0.07 g)
**Hen day egg production (%)**	L4 (65.21^b^ ± 3.73)	L3 (62.32^b^ ± 3.73)	L2 (61.76^ab^ ± 3.81)	L1 (56.27^a^ ± 3.81)
**Hen housed egg production (number)**	L4 (46.30^b^ ± 1.38)	L3 (44.24^b^ ± 1.38)	L2 (42.00^ab^ ± 1.38)	L1 (38.27^a^ ± 1.38)

NB: Please remove vertical lines from all the tables above.

## 4 Discussion

In the current study, four lines of quails were compared to determine which one produced the best egg and meat characteristics. As a result, several measurements were taken and these cumulative measurements may provide a solid foundation for selecting the best egg/meat productive line to meet the needs of the desired breeders.

### 4.1 Body size and weight gain from hatch to fifth weeks of age

The body weight of the different lines of Japanese quails observed a progressive increase from hatch to fifth week of age and the significant differences between lines at different ages were in agreement with the findings of the earlier researchers in Japanese quails in India (Vali et al., 2005; Akram et al., 2014). The differences at all ages of L1, L2, L3, and L4 could be due to genetic variation among them. Body weights of the progeny varied, possibly due to differences in selection strategies for the different lines. The L1, L2, and L3 lines were chosen for heavier body weight, while L4 was chosen for medium body weight. In many other studies, improved body weight in Japanese quail was also observed in birds selected for higher body weight (Akram et al., 2014; Ahmad et al., 2019).

Petek et al. (2003) discovered that hatch weight of chicks produced from smaller eggs was higher than hatch weight of chicks produced from larger eggs, which is similar to the results of this study in the case of line 4. The observed body weights from hatch to fifth week were larger than those of Dauda et al. (2014), who reported body weights of 5.74 ± 1.10 g, 10.88 ± 1.10 g, 23.70 ± 1.18 g, 34.73 ± 1.18 g, 54.54 ± 1.19 g and 76.08 ± 1.20 g, respectively at hatch (day 0), first, second, third, fourth and fifth weeks of age under the humid environment conditions of Central Nigeria. The differences could be due to climatic conditions, and the lines differ from our research. Similarly, Feroz Mohammed et al. (2006) and Taskin Atilla et al. (2017) recorded lower body weight under tropical climatic conditions of India and Turkey, respectively. Krishna and Sahitya Rani (2017), and Sangilimadan and Richard Churchil (2018) also found significantly higher body weight and body weight gain than the results obtained in the current study for the Japanese quails maintained under wet and dry semi-arid climate and hot and humid tropical climatic conditions of India respectively. The variation in body weight between the studies may primarily be due to genetic variation, management procedures, and environmental factors, all of which play a significant role in the growth performance of Japanese quails.

### 4.2 Feed intake and its conversion ratio from hatch to fifth week of age

In the present study, the selection for high and medium body weights resulted in corresponding improvement in feed conversion ratio, and is consistent with the reports of earlier researchers (Khalid and Ali, 2017; Taskin Atilla et al., 2017; Sangilimadan and Richard Churchil, 2018). As a result of their larger bodies, certain birds may consume more feed on a daily and cumulative basis (Khaldari et al., 2010; Akram et al., 2014). The best FCR to a specific body weight may be explained in part by lower maintenance costs and less fat build-up in birds with faster growth rates. The feed conversion ratio observed in different lines were higher in the first week, but Krishna and Sahitya Rani (2017) showed a lower first week feed conversion ratio of 1.89 ± 0.02 to 4.73 ± 0.07 at the sixth week of age in a Japanese quail lines maintained under tropical climatic condition of Telangana, India. Due to differences in the genetic background of the breeds, significant differences in feed intake were observed in different breeds of Japanese quails and varied climatic conditions also played a role in variation between the different studies.

### 4.3 Body weight of females at laying period

The highly significant difference in body weight between the selected lines is in accordance with the earlier reports (Vieira Filho et al., 2016; Dzuriatmono et al., 2019). The mean body weight at seventh week observed in the current study (266.87 g) was higher than the value of 244.10 g by Lofti et al. (2012). Furthermore, Daikwo et al. (2014) and Dauda et al. (2014) both showed significantly lower body weights of 145 ± 0.74 g and 138.91 ± 0.64 g, respectively than those observed in the present study. However, greater than the present weights of 332.33 ± 2.45 and 346.3 ± 2.71 g, respectively, as determined by Arumugam et al. (2011) and Lukanov et al. (2018). The variation in body weight between studies may be primarily due to genetic variation and environmental factors between studies conducted in different locations. The reduction in body weight observed in the 15th week due to increased egg production were in line with those of Ojo et al. (2011), who claimed that body weight appeared to drop as egg production rises.

### 4.4 Age at sexual maturity and egg production characteristics

The age at first egg reported in different lines is very close to the value of 47–53 days reported by other researchers (Ashok and Mahipal Reddy, 2010; Lofti et al., 2012; Daikwo et al., 2014; Dzuriatmono et al., 2019). However, higher values of 54.49 ± 0.20, 55.79 and 64.17 ± 1.17 were reported by Dauda et al. (2014), Krishna and Sahitya Rani (2017) and Arumugam et al. (2011). Lower than the presented estimated value of 44.71 ± 0.62 days was also reported by Hussain et al. (2016) in pure line grand parent stock.

The hen-housed and hen-day egg production performance of Japanese quails in the present study were found to be in agreement with the findings of earlier researchers (Arumugam et al., 2011; Akram et al., 2014; Hussain et al., 2016; Taskin Atilla et al., 2017; Dzuriatmono et al., 2019). Subramaniam (2004) observed much higher mean per cent hen-day egg production of 76.05 ± 2.56 per cent to 85.92 ± 1.51 per cent and hen-housed egg production of 74.19 ± 3.74 to 95.15 ± 0.80 per cent in different lines of Japanese quails maintained under the tropical climatic conditions. Similar results were observed by Subhashini et al. (2018) with mean overall hen day egg production of 79.40 ± 6.00 per cent and Prabakaran and Ezhil Valavan (2020) reported overall hen day egg production and hen-housed egg production (6–46 weeks) of 79.00 ± 1.13 and 76.41 ± 1.31 per cent, respectively. When compared to the current four lines of Japanese quails, Lofti et al. (2012), Dauda et al. (2014), and Krishna and Sahitya Rani (2017) found reduced egg production in Japanese quails maintained at varied climatic conditions of India.

### 4.5 Egg weight from 6th to 16th week of age and feed efficiency

Non-significant to significant differences in egg weight between the lines were also reported by other researchers in Japanese quails in tropical climatic conditions. Ashok and Mahipal Reddy (2010) observed that brown strain exhibited significantly higher egg weight (11.04 ± 0.07 g) than white breasted (10.63 ± 0.06 g) and dark cinnamon brown (10.43 ± 0.02 g) strains, respectively. Genchev (2012) also indicated that egg weight of Pharaoh breed (13.71 ± 0.022 g) was significantly higher than that of the Manchurian golden breed (13.25 ± 0.029 g). However, Sakunthala Devi et al. (2010) observed no significant difference among the black and brown strains for egg weight (14.13 ± 0.10 and 14.32 ± 0.09 g) at 16th weeks of age. The egg weight observed at different ages in the present study are in accordance with Hrnčár et al. (2014), who reported that the average egg weight ranged between 11.48 ± 1.72 g and 13.06 ± 2.05 g, respectively. Similarly, Inci Hakan et al. (2015) reported average egg weight of 11.65 ± 0.14 g, 12.04 ± 0.15 g and 11.96 ± 0.15 g in white, dark brown and golden strains of Japanese quails. Prabakaran and Ezhil Valavan (2020) reported an average egg weight of about 13.12 ± 0.08 g and a value of 13.63 ± 0.34 g was also observed by Subhashini et al. (2018).

The heavy class of birds laid heavier eggs than the other classes combined. Similarly, Jatoi et al. (2013) observed that heavier birds lay heavier eggs at the beginning and end of the productive period. It could be explained by the positive relationship between egg weight and selection for increased body weight and egg number. As a result of this change in allele frequency controlling egg weight, ova size and albumen secretions increase (Altinel et al., 1996; Ahmad et al., 2019). The current study supports the findings of Alkan et al. (2010), who discovered a significant difference in egg weight for both lines chosen for higher body weight and egg production in Japanese quails. El-Deen et al. (2015) also reported increased egg weight after two generations of selection for higher egg production compared to the control line.

Feed consumption during the laying period is influenced not only by body weight, but also by genetics and the time of year (Jatoi et al., 2013; Guimarães et al., 2014). There was a significant difference in feed efficiency per dozen egg in the present study, which is similar to the findings of Arumugam et al. (2011) in pure line Japanese quail breeder birds. Rehman et al. (2022) also reported significant difference in feed per dozen eggs of genetic lines and generations as well as their interaction. Better and increased feed efficiency in selected birds could explain the improved feed efficiency per dozen egg. The current study supports the findings of Kosba et al. (2003), who reported improved FCR in selected lines of Japanese quail compared to the control group. Similarly, another study found that birds selected for higher 4-week body weight in Japanese quail had higher feed efficiency (Khaldari et al., 2010). In another experiment, however, there was no difference in feed efficiency per dozen egg between groups of local and imported Japanese quail (Rehman, 2006). The obtained results in the present study showed lower feed consumption and higher feed efficiency than those reported by Subramaniam (2004) and Akram et al. (2014) in Japanese quails maintained under various tropical conditions in India. Taskin Atilla et al. (2017) found that heavier birds consumed more feed than lighter birds. Similar findings were obtained by Arumugam et al. (2011), Hussain et al. (2016), and Lukanov et al. (2018).

The livability from day-old to 16th week of age was 100 per cent. This is because of selection of quality chicks at the day-old stage itself and standard management practices. Livability standards are also attributed to other environmental factors responsible for the health of the birds along with the purpose of selection. This 100 per cent livability is supported by Almeida et al. (2002), who observed no mortality during the experimental period, and Subramaniam (2004), who found no mortality up to fourth week in L1, L2, L3, and L4. However, Prabakaran and Ezhil Valavan (2020) observed an average livability rate from 0–6 weeks of age of about 94.54 percent and Subhashini et al. (2018) reported overall livability of Japanese quail of 96.00 ± 0.88 percent. For further improving meat production, the traits such as body weight, body weight gain, and feed efficiency may be taken into consideration. For egg production, age (days) at first egg, age at 50%, age at 90% of egg production, and feed efficiency/dozen eggs may be taken into consideration.

## 5 Conclusion

The findings of the current study demonstrated that the growth performance and egg production features of Japanese quails were significantly influenced by pedigree-based selection. There were significant differences between the offspring of the various lines in terms of growth performance or egg production. It can be determined from the present findings that the performance L2 and L3 was better with regards to higher body weight; and this can inform decisions on subsequent genetic improvement programs and also recommend additional research for utilization of L4 for egg production. Based on the favourable overall growth performance, Japanese quails’ production can be widely promoted as a new venture to provide nutritional security and employment to rural youth as an alternative to poultry farming in north-western agroclimatic conditions of Tamil Nadu, India.

## Data Availability

The raw data supporting the conclusions of this article will be made available by the authors, without undue reservation.
